# Association of Elevated Serum GM-CSF, IFN-*γ*, IL-4, and TNF-*α* Concentration with Tobacco Smoke Induced Chronic Obstructive Pulmonary Disease in a South Indian Population

**DOI:** 10.1155/2018/2027856

**Published:** 2018-08-01

**Authors:** Ankita Mitra, Sangeetha Vishweswaraiah, Tania Ahalya Thimraj, Mahendra Maheswarappa, Chaya Sindaghatta Krishnarao, Komarla Sundararaja Lokesh, Jayaraj Biligere Siddaiah, Koustav Ganguly, Mahesh Padukudru Anand

**Affiliations:** ^1^SRM Research Institute, SRM University, Chennai 603203, India; ^2^JSS Medical College and Hospital, JSS University, Department of Pulmonary Medicine, Mysuru, India; ^3^Work Environment Toxicology, Institute of Environmental Medicine, Karolinska Institutet, Box 287, SE-171 77 Stockholm, Sweden

## Abstract

**Background:**

Chronic obstructive pulmonary disease (COPD) is a devastating condition with limited pharmacotherapeutic options and exceptionally high public-health burden globally as well as in India. Tobacco smoking is the primary cause for COPD among men in India. Systemic inflammation involving altered regulation of cytokines controlling the host defense mechanism is a hallmark of COPD pathogenesis. However, biomarker discovery studies are limited among Indian COPD patients.

**Methods:**

We assessed the serum concentrations [median (25th-75th percentile) pg/ml] of interleukin (**IL**)-2,4,6,8,10, granulocyte macrophage colony stimulating factor (**GM-CSF**), interferon gamma (**IFN-γ**), and tumor necrosis factor alpha (**TNF-α**) using a multiplexed immunoassay. Our study cohort consisted of 30 tobacco smokers with COPD (**TS COPD**) and 20 tobacco smokers without COPD (**TS CONTROL**) from South India. The study population was matched for age, sex (male), and tobacco consumption (pack-years). COPD was diagnosed according to the global initiative for chronic obstructive lung disease (GOLD) criteria of persistent airflow obstruction determined by the ratio of postbronchodilator forced expiratory volume in 1 second/forced vital capacity (FEV_1_/FVC) of <0.7. A validated structured questionnaire-based survey [Burden of Obstructive Lung Disease (BOLD) study] and spirometry were performed during house to house visit of the field study. Statistical analysis included nonparametric (two-tailed) Mann–Whitney U and Spearman rank test, as appropriate (significance: p<0.05).

**Results:**

Serum GM-CSF [69.64 (46.67, 97.48); 36.78 (30.07, 53.88), p=0.014], IFN-*γ* [51.06 (17.00, 84.86); 11.70 (3.18, 32.81), p=0.017], IL-4 [9.09 (1.8, 19.9); 1.8 (1.8, 4.46); p=0.024], and TNF-*α* [20.68 (5.5, 29.26); 3.5 (3.5, 4.5); p<0.001] concentrations (pg/ml) were increased in TS COPD subjects compared to TS CONTROL. A weak correlation between lung function parameters and cytokine concentrations was detected.

**Conclusion:**

Our pilot study reveals GM-CSF, IFN-*γ*, IL-4, and TNF-*α* as plausible COPD susceptibility biomarkers within the investigated South Indian population that needs to be validated in a larger cohort.

## 1. Introduction

Chronic obstructive pulmonary disease (COPD), the 4th leading cause of death worldwide [1], is characterized by persistent airflow obstruction. Conservative estimates suggest that approximately 30 million people in India currently suffer from COPD [2, 3], which in turn represents the prevalence of the devastating disease and socioeconomic burden. Tobacco smoking is the main risk factor for development of COPD. Other environmental exposures including biomass smoke and air pollution also contribute to the pathogenesis of COPD [4–7]. Tobacco smoke induced COPD is predominant among men in India [8]. The global initiative for chronic obstructive lung disease (GOLD) defines COPD as a “common, preventable and treatable disease characterized by persistent respiratory symptoms and airflow limitation due to airway and/or alveolar abnormalities usually caused by significant exposure to noxious particles or gases” [3, 9]. Clinically COPD is diagnosed as a persistent airflow obstruction determined by the ratio of postbronchodilator forced expiratory volume in 1 second/forced vital capacity (FEV_1_/FVC) of <70% and an FEV_1_ of <80% [3].

Altered host defense mechanism is a hallmark of COPD that is characterized by local as well as systemic alterations of chemokine and cytokine regulation. It is suggested that the inflammatory process originates in the airways and lung parenchyma in COPD which then spills over into the systemic circulation [10–13]. However, correlation between lung inflammation and systemic inflammation as well as the corresponding cytokine balance in COPD has not been consistently observed [14, 15]. It is also considered that the systemic inflammation in COPD might be primarily driven by tobacco smoking as observed in case of cardiovascular diseases [10, 16]. Several proinflammatory cytokines, T-cell cytokines, growth factors, chemokines, and anti-inflammatory cytokines have been implicated in COPD pathogenesis [17, 18]. For example, Interleukin 6 (IL-6) and tumor necrosis factor alpha (TNF-*α*) enhance COPD by increasing inflammation [17, 19–21]. Significantly higher levels of IL-2 have been reported in the plasma of patients with stable COPD compared to rapidly progressing COPD and may influence disease progression [22]. IL-4 might enhance COPD by increasing the production of IgE [23–25]. Granulocyte macrophage colony stimulating factor (GM-CSF) also contributes to the enhancement of COPD through increasing the number of neutrophils [26, 27]. Increased levels of IL-8 play a pivotal role in COPD pathogenesis by inducing and sustaining inflammatory response as it is a chemoattractant for neutrophils [28, 29]. Interferon gamma (IFN-*γ*) decreases the number of Th2 cells thereby enhancing COPD [30]. Increased levels of the anti-inflammatory cytokine IL-10 have been associated with reduced COPD exacerbations [31–35]. Thus, it becomes important to assess the systemic and local chemokine and cytokine expression among well-characterized COPD subjects with similar socioeconomic status, geographical location, ethnicity, and exposure particularly in case of developing countries like India which represents enormous diversity. The “Mysuru studies of Determinants of Health in Rural Adults (MUDHRA)” in India is an ongoing project taking into consideration the above-mentioned factors to phenotype COPD patients in South India [36–38]. However, biomarker discovery studies along with addressing the above-mentioned cohort characterization approach are limited in India. Therefore in this pilot study, we assessed the cytokine concentrations of IL-2, 4, 6, 8, 10, GM-CSF, IFN-*γ*, and TNF-*α* using a panel assay in a rural cohort of long-term tobacco smokers with and without COPD. The concentrations of cytokines exhibiting significant difference were further analyzed for correlation to lung function parameters to identify plausible systemic biomarkers for COPD in the study cohort.

## 2. Materials and Methods

### 2.1. Subject Population

The cohort consisted of 30 tobacco smokers with COPD (**TS COPD**; moderate: 14, severe: 12, and very severe: 4) and 20 tobacco smokers without COPD (**TS CONTROL**) dwelling in the same geographical area (Mysuru, Karnataka, India). All subjects were male and current smokers [except 3 subjects in the TS COPD group who quitted smoking 3, 8, and 10 years ago at ages (y) 67, 55, and 62, respectively] and reported chronic bronchitis. The tobacco consumption (pack-years) was matched between the TS COPD and TS CONTROL groups. COPD was diagnosed according to the global initiative for chronic obstructive lung disease (GOLD) criteria of persistent airflow obstruction determined by the ratio of FEV_1_/FVC of <0.7 and the severity of COPD was classified according to GOLD guidelines. A detailed validated questionnaire based on the burden of obstructive lung disease (BOLD) study translated in regional language (Kannada) was used to assess the exposure scenario and other background information following a house to house visit by trained field workers who had earlier participated in the urban Mysore BOLD study and trained in the BOLD protocol.

All subjects were in stable condition and without any reported infection in the previous 4 weeks prior to blood sampling. Subjects with histories of any other respiratory disease like asthma and tuberculosis were excluded. Chest X-ray and sputum for acid-fast staining were done for most chest symptomatics to rule out tuberculosis. Subjects with any reported cardiovascular and metabolic diseases such as diabetes were also excluded. Most of the patients were naïve to inhaled corticosteroid/long-acting beta-agonist inhalers (ICS/LABA) and not on any regular medications. They used medications only for managing the intermittent acute exacerbations. This scenario is common in rural India. The demographics of the study cohort are provided in Table 1. The cohort characterization procedure has been previously described [36–38].

3 ml of venous blood samples was collected from each subject by trained professionals using the venipuncture method in the BD Vacutainer® PLUS plastic serum tubes with spray-coated silica. The tubes were incubated in an upright position at room temperature for 30 minutes and centrifuged for 15 minutes at 2500 RPM. The supernatant (serum) was carefully aspirated without disturbing the cell layer into prelabeled cryovials and stored at –80°C till further use. All procedures of this study were approved by Institutional Ethical Clearance JSS Medical College (JSSMC/IEC/13/4048/2016-2017), Mysore, Karnataka, India. Informed and written consent was obtained from each subject.

### 2.2. Cytokine Panel Assay

Concentrations of eight proinflammatory cytokines, IL-2, 4, 6, 8, 10, GM-CSF, IFN-*γ*, and TNF-*α* were measured using the Bio-Plex Pro Assays 8-Plex assay kit (Bio-Rad, Cat#M50-0007A) and Bio-Plex Multiplex immunoassay system (Biorad Bio-Plex 200) according to the manufacturer's instruction. Bio-Plex Manager™ and Bio-Plex Data Pro™ Software were used to analyze the generated multiplex data. All samples were measured in duplicate and the mean of the two measurements was considered. The assay passed the quality control of the manufacturer. Concentrations of cytokines are presented as median (25th-75th percentile) pg/ml. Out of range (<OOR) values were considered as the lowest detectable value. There were no >OOR values. Supplementary Table ST1 shows the number of <OOR values for each analyte along with the lowest detectable concentration.

Briefly, diluted 50*μ*l of (1X) magnetic beads was added to individual wells of a 96-well plate and adhered using vacuum filtration. After washing, 50*μ*l of prediluted standards and serum samples were added to the respective wells and plate was incubated on a shaker at 850 rpm for 45 min at room temperature. Thereafter, the plate was washed and 25*μ*l prediluted Bio-Plex (1X) detection antibody was added and incubated for 30 minutes. After incubation followed by washing with Bio-Plex assay buffer, 50*μ*l prediluted (1X) streptavidin-conjugated PE was added and incubated for 10 minutes followed by an additional wash with Bio-Plex assay buffer and then 125*μ*l assay buffer was added. Thereafter, the plate was placed for incubation on shaker for 5 minutes at 850 rpm. Then the plate was analyzed using the Bio-Plex Protein Array system and the concentrations of each cytokine were determined using software Bio-Plex 200 based on Luminex technology.

### 2.3. Statistics

Nonparametric Mann–Whitney U (two-tailed) t-test was performed to determine the statistical significance for the difference of cytokine concentrations between the TS COPD and TS CONTROL groups. Two-tailed nonparametric Spearman rank test was used to analyze the correlation with FEV_1-pre_, FEV_1-post_, FEV_1-post_/FVC, and pack-years with cytokine concentrations. The correlation study was performed only with the significantly different cytokines (GM-CSF, IFN-*γ*, IL-4, and TNF-*α*) among TS COPD and TS CONTROL groups. p<0.05 was considered to be statistically significant. All statistical analyses were performed using GraphPad Prism software (Version: 5) (La Jolla, California).

### 2.4. Results

Concentrations of GM-CSF, IFN-*γ*, IL-4, and TNF-*α* were significantly different among the eight cytokines measured between TS COPD and TS CONTROL in our study cohort (Table 2). The correlation between cytokine concentrations and lung function parameters was weak. Concentrations of significantly different cytokines were not correlated to tobacco consumption (pack-years).

### 2.5. GM-CSF

**Figure 1:**
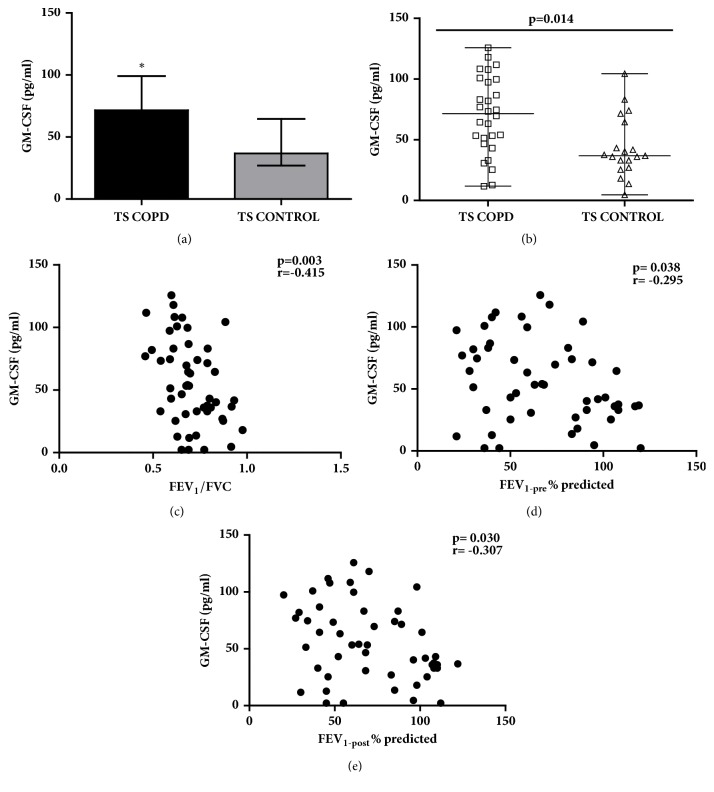
**Serum concentrations of granular macrophage colony stimulating factor (GM-CSF) and corresponding correlation with lung function parameters among long-term smokers with or without chronic obstructive pulmonary disease (COPD)**. (a, b) GM-CSF concentrations were increased in TS COPD group compared to TS CONTROL group (p=0.014); inverse correlation of GM-CSF concentrations to (c) FEV_1_/FVC and (d) FEV_1(pre)_ and FEV_1(post)_ was detected in the study cohort. Data is presented as median (25th-75th percentile); TS COPD: n=30 and TS CONTROL: n=20; *∗*p<0.05 was considered as statistically significant.** COPD**: chronic obstructive pulmonary disease;** FEV**_1_**/FVC**: forced expiratory volume 1 second /forced vital capacity; **FEV**_1(pre)_: forced expiratory volume 1 second prebronchodilator challenge; **FEV**_1  (post)_: FEV_1_ postbronchodilator challenge;** TS COPD**: tobacco smokers with COPD;** TS CONTROL**: tobacco smokers without COPD.

Increased GM-CSF concentration (Figures 1(a) and 1(b)) has been detected in TS COPD subjects compared to TS CONTROL in our study cohort [TS COPD: 69.64 (46.67, 97.48); TS CONTROL: 36.78 (30.07, 53.88) pg/ml; p=0.014]. The GM-CSF concentrations correlated inversely with the lung function parameters FEV_1_/FVC (p=0.003; r=-0.415; Figure 1(c)), FEV_1-pre_ (p=0.038; r=-0.295; Figure 1(d)), and FEV_1-post_ (p=0.030; r=-0.307; Figure 1(e)).

### 2.6. IFN-*γ*

**Figure 2:**
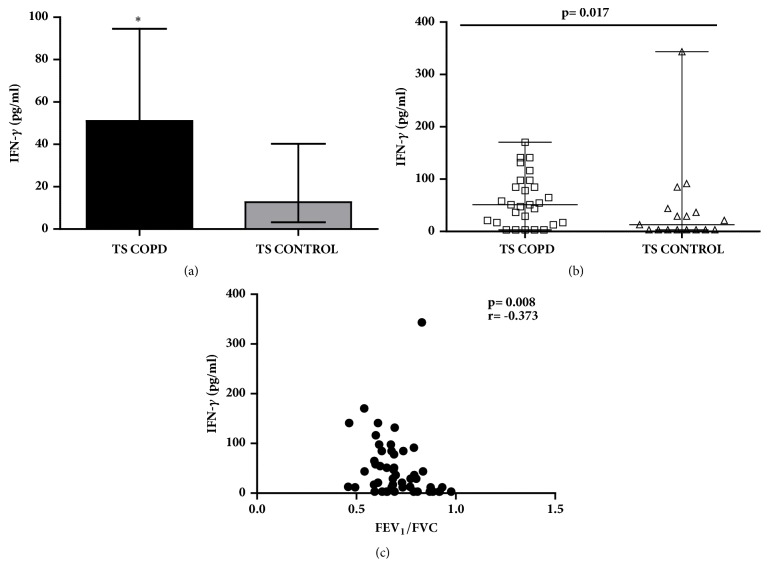
**Serum concentrations of interferon gamma (IFN-** **γ** **) and corresponding correlation with lung function parameters among long-term smokers with or without chronic obstructive pulmonary disease (COPD)**. (a, b) IFN-*γ* concentrations were increased in TS COPD group compared to TS CONTROL group (p=0.017); inverse correlation of IFN-*γ* concentrations to (c) FEV_1_/FVC was detected in the study cohort. *∗*p<0.05 was considered as statistically significant. Data is presented as median (25th-75th percentile); TS COPD: n=30 and TS CONTROL: n=20; *∗*p<0.05 was considered as statistically significant.** COPD**: chronic obstructive pulmonary disease;** FEV**_1_**/FVC**: forced expiratory volume 1 second postbronchodilator challenge/forced vital capacity; **FEV**_1(pre)_: forced expiratory volume 1 second prebronchodilator challenge; **FEV**_1  (post)_: FEV_1_ postbronchodilator challenge;** TS COPD**: tobacco smokers with COPD;** TS CONTROL**: tobacco smokers without COPD.

The IFN-*γ* concentrations (Figures 2(a) and 2(b)) were increased in TS COPD subjects compared to TS CONTROL in our study cohort [TS COPD: 51.06 (17.00, 84.86); TS CONTROL: 11.70 (3.18, 32.81) pg/ml; p=0.017]. The IFN-*γ* concentrations correlated inversely with the lung function parameter FEV_1_/FVC (p=0.008; r=-0.373; Figure 2(c)).

### 2.7. IL-4

**Figure 3:**
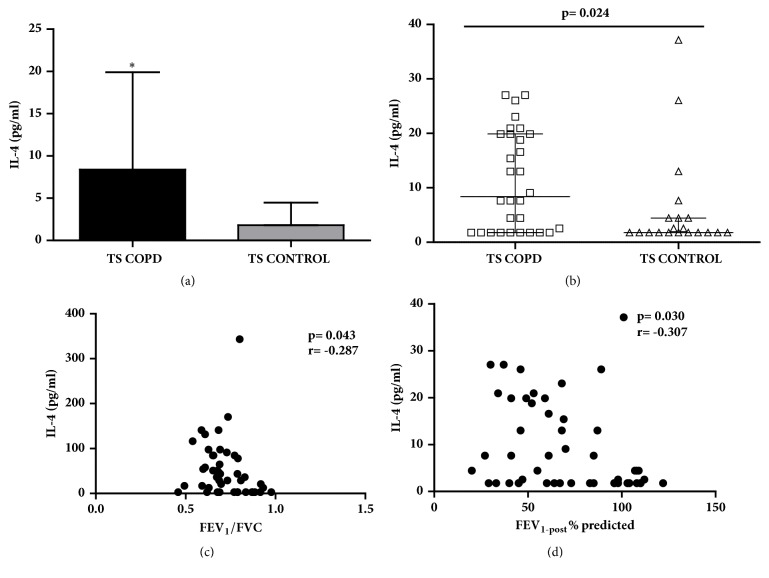
**Serum concentrations of interleukin 4 (IL-4) and corresponding correlation with lung function parameters among long-term smokers with or without chronic obstructive pulmonary disease (COPD)**. (a, b) IL-4 concentrations were increased in TS COPD group compared to TS CONTROL group (p=0.024); inverse correlation of IL4 concentrations to (c) FEV_1_/FVC and (d) FEV_1(post)_ was detected in the study cohort.. Data is presented as median (25th-75th percentile); TS COPD: n=30 and TS CONTROL: n=20; *∗*p<0.05 was considered as statistically significant.** COPD**: chronic obstructive pulmonary disease; **FEV**_1(pre)_: forced expiratory volume 1 second prebronchodilator challenge; **FEV**_1  (post)_: FEV_1_ postbronchodilator challenge;** TS COPD**: tobacco smokers with COPD;** TS CONTROL**: tobacco smokers without COPD.

Increased IL-4 concentrations (Figures 3(a) and 3(b)) have been detected in TS COPD subjects compared to TS CONTROL in our study cohort [TS COPD: 9.09 (1.8, 19.9); TS CONTROL: 1.8 (1.8, 4.46) pg/ml; p=0.024]. The IL-4 concentrations correlated inversely with the lung function parameters FEV_1_/FVC (p=0.043; r=-0.287; Figure 3(c)) and FEV_1-post_ (p=0.030; r=-0.307; Figure 3(d)).

### 2.8. TNF-*α*

**Figure 4:**
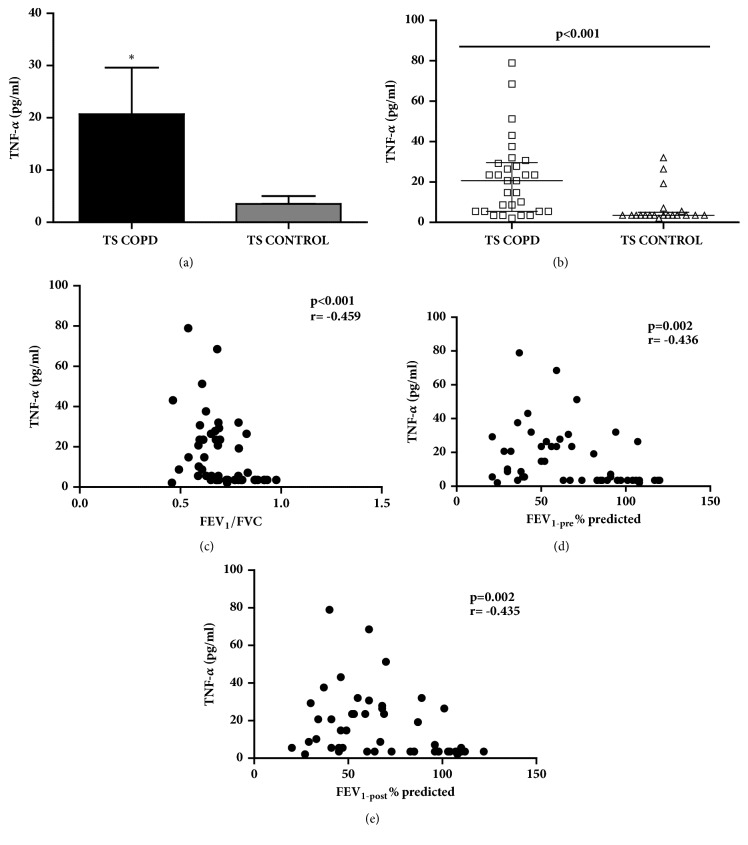
**Serum concentrations of tumor necrosis factor alpha (TNF-** **α** **) and corresponding correlation with lung function parameters among long-term smokers with or without chronic obstructive pulmonary disease (COPD)**. (a, b) TNF-*α* concentrations were increased in TS COPD group compared to TS CONTROL group (p<0.001); inverse correlation of TNF-*α* concentrations to (c) FEV_1_/FVC, (d) FEV_1(pre)_, and (e) FEV_1(post)_ was detected in the study cohort. Data is presented as median (25th-75th percentile); TS COPD: n=30 and TS CONTROL: n=20; *∗*p<0.05 was considered as statistically significant.** COPD**: chronic obstructive pulmonary disease;** FEV**_1_**/FVC**: forced expiratory volume 1 second postbronchodilator challenge/forced vital capacity; **FEV**_1(pre)_: forced expiratory volume 1 second prebronchodilator challenge; **FEV**_1  (post)_: FEV_1_ postbronchodilator challenge;** TS COPD**: tobacco smokers with COPD;** TS CONTROL**: tobacco smokers without.

Increased TNF-*α* concentrations (Figures 4(a) and 4(b)) were detected in TS COPD subjects compared to TS CONTROL in our study cohort [TS COPD: 20.68 (5.5, 29.26); TS CONTROL: 3.5 (3.5, 4.5) pg/ml; p<0.001]. The TNF-*α* concentrations correlated inversely with the lung function parameters FEV_1_/FVC (p<0.001; r=-0.459; Figure 4(c)), FEV_1-pre_ (p=0.002; r=-0.436; Figure 4(d)), and FEV_1-post_ (p=0.002; r=-0.435; Figure 4(e)).

## 3. Discussion

Our study included subjects recruited from two villages near Mysuru, Karnataka, India, with similar socioeconomic status and ethnicity. We have been performing COPD phenotyping studies in the same region for the study titled “Mysuru studies of Determinants of Health in Rural Adults (MUDHRA)” [35, 36]. A total of 8457 men and women above the age of 30 years from randomly selected 16 villages in rural Mysore district were screened for chronic respiratory diseases. 1692 subjects were invited to participate in pre- and postbronchodilator spirometry. 1269 participated and 1085 satisfied American Thoracic Society (ATS) criteria. These 1085 subjects make up the MUDHRA cohort and have since been followed up twice with repeat spirometry to monitor the decline in lung functions. The cases and controls in this study were from this cohort. The Indian subcontinent offers a remarkable diversity in regard to ethnicity of people as well as their socioeconomic status. Therefore, in order to perform biomarker discovery studies, consideration of the above-mentioned factors becomes crucial. All the eligible subjects answered a validated demographic and clinical questionnaire. Age and gender matched controls underwent pre- and postbronchodilator spirometry (acceptable spirometry: ATS). Due to cultural reasons, tobacco smoke induced COPD is particularly observed among men in India and most women in rural India do not smoke. Thus, our study was confined to males only. One of the important strengths of the two groups (TS COPD and TS CONTROL) is the matched tobacco consumption, age, socioeconomic status, and dwelling habits. This provided a strong base to carry out our pilot study investigating the systemic concentrations of IL-2, 4, 6, 8, 10, GM-CSF, IFN-*γ*, and TNF-*α* within the two groups. Our analysis identified serum concentrations of GM-CSF, IFN-*γ*, IL-4, and TNF-*α* to be significantly increased among TS COPD group compared to TS CONTROL. Even though IL-2, 6, 8, and 10 have been consistently implicated in COPD pathogenesis, similar tobacco consumption among TS COPD and TS CONTROL groups in our study cohort may explain the lack of any difference in their levels. This also highlights the fact that systemic GM-CSF, IFN-*γ*, IL-4, and TNF-*α* level alone or in combination may play a crucial role in determining susceptibility for COPD onset among individuals with similar tobacco load in our study cohort. Correlation of GM-CSF, IFN-*γ*, IL-4, and TNF-*α* concentrations to FEV_1-pre_, FEV_1-post_, and/ or FEV_1_/FVC, although weak, further supports our finding. However, we recognize the fact that the generated data on the investigated cytokines from the cell free bronchoalveolar lavage (BAL) fluid induced sputum and/ or airway biopsies along with BAL and blood differential cell counts; fractional exhaled nitric oxide (FeNO) and arterial oxygen saturation levels would have improved the understanding of our findings.

GM-CSF is a key regulator for the differentiation and survival of neutrophils, eosinophils, and macrophages and has been implicated in COPD [18]. It is released by alveolar macrophages of COPD patients [40] and increased concentrations in BAL fluid of patients with COPD are correlated with the increased numbers of neutrophils [27]. Increased systemic GM-CSF levels have been reported in subjects exposed to high levels of particulate matter (PM) particularly PM10 [41, 42]. Tobacco smoke exposed alveolar macrophages produce GM-CSF along with other cytokines that induce the proliferation and release of polymorphonuclear leucocytes and monocytes from the bone marrow [43]. The studies showed that a range of different PMs stimulate alveolar macrophages to produce proinflammatory cytokines, and these cytokines are also increased in the blood of subjects during acute air pollution episodes. This indicates a temporal link between these cytokines and the systemic response. Interestingly, it has been shown that GM-CSF itself is a weak or ineffective direct inducer of inflammatory cytokine production in cells other than macrophages or neutrophils [44]. However, it exerts its effect by increasing cytokine gene expression [45–47] and in particular priming inflammatory cells to amplify their responses to other stimuli such as IFN*γ* [48, 49]. Oudijk et al. [50] reported that progression of COPD to be associated with the activation of neutrophils following cytokine gene expression studies in GM-CSF stimulated peripheral blood neutrophils of healthy subjects and COPD patients.

IFN-*γ* secreting T cells (Th1, Tc1) are increased in the airways of COPD patients [49]. IFN-*γ* orchestrates the infiltration of Th1 and Tc1 cells in the COPD lungs through the upregulation of the chemokine receptor, CXC-chemokine receptor (CXCR) 3, on these cells and the release of the CXCR3-activating chemokines [51]. Consistent with Th1 responses, IFN-*γ* levels are elevated in COPD. IFN-*γ* has been also associated with the development of emphysema in mice [52, 53]. Moermans et al. [54] demonstrated increased release of IFN-*γ* from the blood leukocytes of COPD patients compared to those of healthy subjects. IFN-*γ* was detectable in 61% of COPD and only 27% of healthy subjects. Interestingly, 75% of COPD patients producing IFN-*γ* from their sputum cells also released IFN-*γ* from their blood cells. The enhanced release of IFN-*γ* in COPD patients supported the role Th1 pathway at the local as well as systemic level.

IL-4 is a Th2 cytokine that regulates the differentiation of uncommitted T0 cells to Th2 cells. It also plays an important role in the isotype switching of B cells from IgG to IgE producers [17]. IL-4 expression is increased in the CD8^+^ cells (Tc2 cells) of bronchoalveolar lavage of COPD patients [18, 24]. Several studies reported the increase of TNF-*α* concentrations in the sputum and plasma of COPD patients during acute exacerbations. Increased systemic TNF-*α* level has been detected in COPD patients with weight loss due to its secretion from circulating cells [18, 52, 55]. This has been also supported by mouse studies where mice overexpressing pulmonary TNF-*α* resulting in emphysema also exhibited increased plasma TNF-*α* and muscle wasting [56]. Recently, increased serum TNF-*α* level has been also correlated to the severity of airway obstruction among COPD patients [57].

### 3.1. Conclusions

Our findings of increased serum GM-CSF, IFN-*γ*, IL-4, and TNF-*α* concentrations are consistent with the overall understanding of COPD not only as a lung inflammatory disorder but also as a systemic inflammatory disorder. Oxidative stress, activation of circulating inflammatory cells, and increased levels of circulating inflammatory cytokines are an integral part of the overall phenomenon [54, 58]. It is plausible that systemic GM-CSF, IFN-*γ*, IL-4, and TNF-*α* level may serve as COPD predisposition markers among long-term smokers in the investigated population that warrants further studies in larger and multiple independent cohorts.

## Figures and Tables

**Table 1:** Demographics of the study cohort constituting tobacco smokers with chronic obstructive pulmonary disease (TS COPD) and tobacco smokers without COPD (TS CONTROL). Values are presented as ^#^median (25th–75th percentile) and ^∧^ frequency (percentage). ^§^Only 3 subjects in the TS COPD group quitted smoking 3, 8, and 10 years ago; *∗* p<0.05 is considered as significant.

	**TS COPD**	**TS CONTROL **	**p value**
**Number of subjects**	30	20	-

**Gender **	Male	Male	-
**A** **g** **e** ^#^	61 (53.0, 68.0)	58 (49.0, 59.25)	0.207

**CCI [39]**	3 (2, 4)	1 (0.5, 2)	<0.0001*∗*
FEV_1 (Pre)_^#^ (L)	1.30 (0.91, 1.77)	2.77 (2.1, 3.11)	0.0001*∗*
FEV_1 (Post)_^#^ (L)	1.44 (0.96, 1.84)	2.83 (2.16, 3.24 )	0.0001*∗*
FVC_(Pre)_^#^ (L)	2.15 (1.67, 2.83)	3.30 (2.59, 4.14)	0.0022*∗*
FVC_(Post)_^ #^ (L)	2.24 (1.49, 1.81)	3.45 (2.44, 3.92)	0.0022*∗*
**FEV**_**1**_ ** % predicted (Pre)** ^** #**^	43 (36.0, 59.0)	96 (88.25, 107.25)	<0.0001
**FEV**_**1**_ ** % predicted (Post)** ^** #**^	48 (40.25, 61.0)	102 (94.25, 108.25)	<0.0001
**FEV**_**1**_ **/FVC** ^**#**^	0.63 (0.59, 0.68)	0.80 (0.78, 0.88)	<0.0001
**Pack-years** ^**#**^	19.1 (13.94, 28.61)	20.9 (18.2, 24.6)	0.751
Ex-smokers ^∧§^	3 (10%)	0 (0%)	-
**Symptom Years** ^**#**^	5 (3, 7)	-	-
**SLI** ^**#**^	19.5 (17, 22)	19 (16, 22)	0.67

**Indoor Animals** ^∧^	17 (56%)	14 (70%)	0.34

**House with mud walls** ^∧^	19 (63.3)	12 (60%)	0.81

**Owns Land** ^∧^	15 (50%)	12 (60%)	0.49

**Owns Vehicle** ^∧^	10 (66.7%)	5 (33.3%)	0.53

**Farmer** ^∧^	11 (36.7%)	5 (25%)	0.48

**Manual labour** ^∧^	15 (50%)	14 (70%)	0.48

**Literate** ^∧^	22 (73.3%)	15 (75%)	0.51

**CCI**: Charlson comorbidity index; **FEV**_1  (Pre)_: forced expiratory volume 1 second prebronchodilator challenge; **FEV**_1  (Post)_: FEV_1_ postbronchodilator challenge; **FVC**: forced vital capacity; **SLI**: standard of living index;** TS COPD**: tobacco smokers with COPD; **TS CONTROL**: tobacco smokers without COPD.

**Table 2:** Serum concentrations [median (25th–75th percentile) pg/ml] of the eight cytokines measured in tobacco smokers with chronic obstructive pulmonary disease (TS-COPD) and TS CONTROL subjects. *∗* p<0.05 is considered as significant.

**Cytokines**	**TS-COPD (n=30)**	**TS-CONTROL (n=20)**	**p value**
	(pg/ml)	(pg/ml)	
**GM-CSF**	69.64 (46.67 - 97.48)	36.78 (30.07 - 53.88)	*∗*0.014

**IFN-** **γ**	51.06 (17 - 84.86)	11.7 (3.18 - 32.81)	*∗*0.017

**IL-2**	2.29 (2.29 - 2.73)	2.29 (2.29 – 2.29)	0.08

**IL-4**	9.09 (1.8 - 19.9)	1.8 (1.8 - 4.46)	*∗*0.024

**IL-6**	14.42 (7.76 - 21.18)	13.04 (11.65-21.34)	0.68

**IL-8**	14.22 (8.96 - 21.23)	22.7 (9.75 – 35.18)	0.40

**IL-10**	3.4 (3.4 - 6.65)	3.6 (2.19 - 6.4)	0.70

**TNF-** **α**	20.68 (5.5 – 29.26)	3.5 (3.5 - 4.5)	*∗*<0.001

**GM-CSF**: granular macrophage colony stimulating factor; **IFN-γ**: interferon gamma; **IL**: interleukin; **TNF-α**: tumor necrosis factor alpha.

## Data Availability

Individual data and range have been provided in the figures for clarity.

## Supplementary Materials

Supplementary Materials Supplementary Table ST1 lists for each analyte the number of OOR values along with the lowest detectable concentration (pg/ml).
