# Supplementary material for: Association of Elevated Serum GM-CSF, IFN-γ, IL-4, and TNF-α Concentration with Tobacco Smoke Induced Chronic Obstructive Pulmonary Disease in a South Indian Population
Source: Int J Inflam. 2018 Aug 1;2018:2027856. doi: 10.1155/2018/2027856 (PMC6092978; doi:10.1155/2018/2027856)
Supplement: Supplementary Materials — Supplementary Table ST1 lists for each analyte the number of OOR values along with the lowest detectable concentration (pg/ml). [file 2027856.f1.docx]

**Supplementary Material**

**Association of elevated serum GM-CSF, IFN-γ, IL-4 and TNF-α concentration with tobacco smoke induced chronic obstructive pulmonary disease in a South Indian population**

Ankita Mitra^1^, Sangeetha Vishweswaraiah^1^, Tania Ahalya Thimraj^1^, Mahendra Maheswarappa^2^, Chaya Sindaghatta Krishnarao ^2^, Komarla Sundararaja Lokesh ^2^, Jayaraj Biligere Siddaiah ^2^, Koustav Ganguly^1,3*^, Mahesh Padukudru Anand ^2*^

^1.^SRM Research Institute, SRM University, Chennai, 603203, India

^2.^JSS Medical College and Hospital, JSS University, Department of Pulmonary Medicine, Mysuru, India

^3^ Work Environment Toxicology; Institute of Environmental Medicine, Karolinska Institutet; Box
287, SE-171 77 Stockholm, Sweden

*** Contributed equally and corresponding authors**

| **Name** | **Email** |
| --- | --- |
| Ankita Mitra | ankitamitra.srm@gmail.com |
| Sangeetha Vishweswaraiah | sangeethav.srm@gmail.com |
| Tania A Thimraj | taniathimraj.srm@gmail.com |
| Mahendra M | mahesims@gmail.com |
| Chaya SK | chaya.sindaghatta@gmail.com |
| Lokesh KS | kslokesh@gmail.com |
| Jayaraj BS | drjayarajbs@yahoo.com |
| Koustav Ganguly | koustav.ganguly@ki.se |
| Mahesh PA | mahesh1971in@yahoo.com |

**Correspondence:**

**Mahesh PA**

Department of Pulmonary Medicine, JSS Medical College and Hospital, JSS University, Shivarathreeshwara Nagar, Mysuru, 570015, **Email:** mahesh1971in@yahoo.com; India. Phone: +91-9448044003

**Koustav Ganguly**

Work Environment Toxicology; Institute of Environmental Medicine, Karolinska Institutet; Box
287, SE-171 77 Stockholm, Sweden; **Email:** koustav.ganguly@ki.se; Phone: +46-0852487133

| **Analyte** | **TS-COPD** | **TS-CONTROL** | **<OOR set as LDL**  **(pg/ml)** |
| --- | --- | --- | --- |
| **GM-CSF** | 2 | 1 | 2.3 |
| **IFN-γ** | 2 | 3 | 11.7 |
| **IL-2** | 20 | 10 | 2.29 |
| **IL-4** | 9 | 11 | 1.8 |
| **IL-6** | 1 | 0 | 1.3 |
| **IL-8** | 1 | 1 | 1.5 |
| **IL-10** | 9 | 2 | 3.4 |
| **TNF-α** | 4 | 14 | 3.5 |

Supplementary table ST1 shows the number of <OOR values for each analyte that were assigned the lowest detectable limit (LDL) for non-parametric ranking.

**Abbreviations:**

**GM-CSF**: granular macrophage colony stimulating factor; **IFN-γ**: interferon gamma; **IL**: interleukin; **TNF-α**: tumor necrosis factor alpha; **TS COPD**: tobacco smokers with COPD; **TS CONTROL**: tobacco smokers without COPD; **OOR**: out of range; **LDL**: lowest detectable limit
